# High genomic diversity of multi-drug resistant wastewater *Escherichia coli*

**DOI:** 10.1038/s41598-018-27292-6

**Published:** 2018-06-12

**Authors:** Norhan Mahfouz, Serena Caucci, Eric Achatz, Torsten Semmler, Sebastian Guenther, Thomas U. Berendonk, Michael Schroeder

**Affiliations:** 1 Biotec, TU Dresden, Dresden, Germany; 2 Institute for Hydrobiology, TU Dresden, Dresden, Germany; 3 United Nations University Institute for Integrated Management of Material Fluxes and of Resources, Dresden, Germany; 4 Institute of Microbiology und Epizootics, FU, Berlin, Germany; 5 Institut für Pharmazie Pharmazeutische Biologie, Ernst-Moritz-Arndt-Universität Greifswald, Greifswald, Germany

## Abstract

Wastewater treatment plants play an important role in the emergence of antibiotic resistance. They provide a hot spot for exchange of resistance within and between species. Here, we analyse and quantify the genomic diversity of the indicator *Escherichia coli* in a German wastewater treatment plant and we relate it to isolates’ antibiotic resistance. Our results show a surprisingly large pan-genome, which mirrors how rich an environment a treatment plant is. We link the genomic analysis to a phenotypic resistance screen and pinpoint genomic hot spots, which correlate with a resistance phenotype. Besides well-known resistance genes, this forward genomics approach generates many novel genes, which correlated with resistance and which are partly completely unknown. A surprising overall finding of our analyses is that we do not see any difference in resistance and pan genome size between isolates taken from the inflow of the treatment plant and from the outflow. This means that while treatment plants reduce the amount of bacteria released into the environment, they do not reduce the potential for antibiotic resistance of these bacteria.

## Introduction

**Figure 1:**
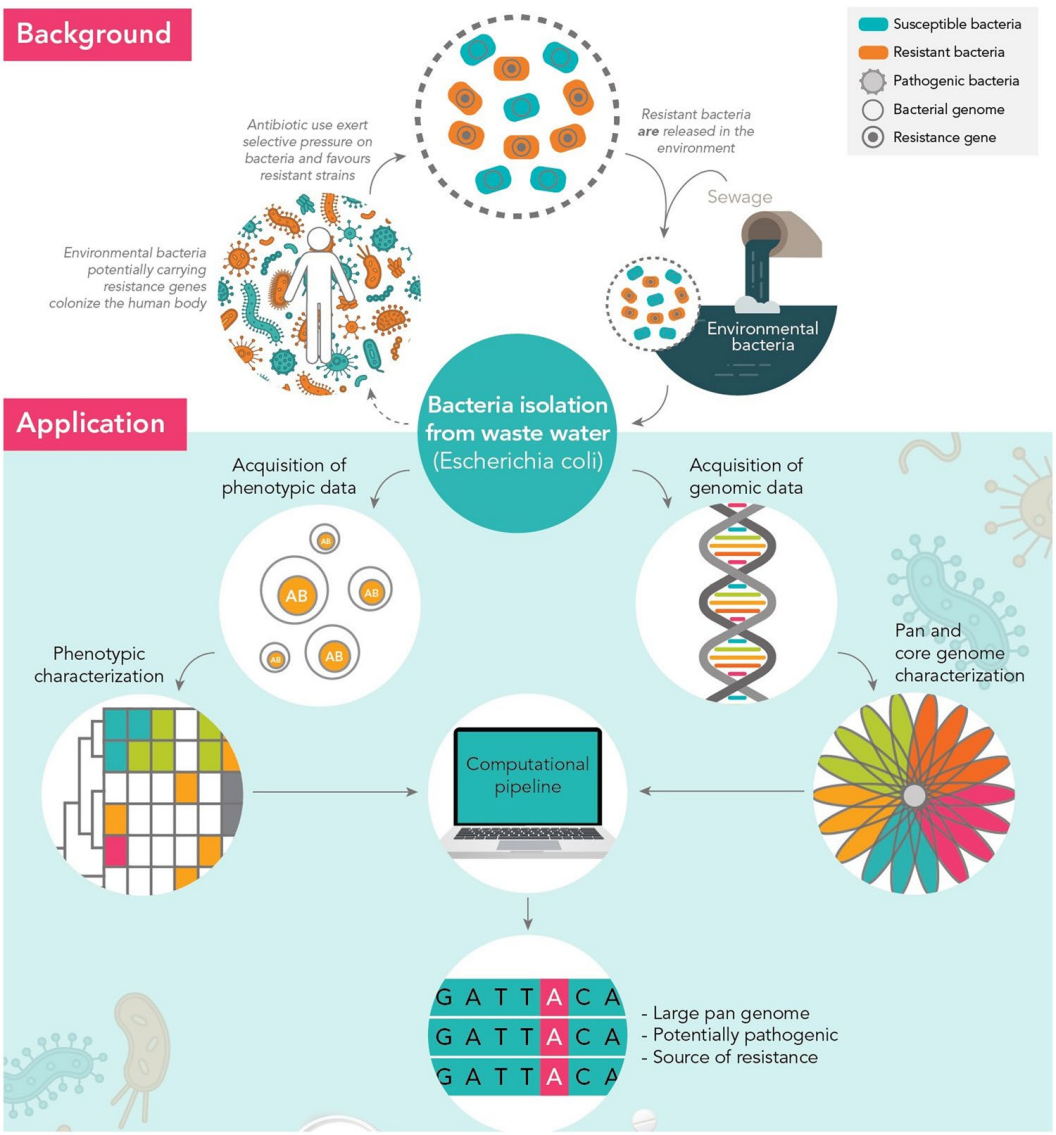
Wastewater plays an important role in antibiotic resistance development. Wastewater *Escherichia coli* isolates were tested for antibiotic resistance and sequenced. Many isolates are multi-drug resistant and have markers often found in pathogenic isolates. Their large pan-genome is a source of potentially novel resistance genes.

In 1945, Alexander Fleming, the discoverer of Penicillin, warned of antibiotic resistance. Today, the WHO echoes this warning, calling antibiotic resistance a global threat to human health. Humans are at the center of the modern rise of resistance. The human gut^1^, clinical samples^2,3^, soil^4,5^, and wastewater^6^ all harbor resistant bacteria and resistance genes. At the heart of modern resistance development is a human-centered network of clinics, industry, private homes, farming, and wastewater. Recent studies suggest that wastewater contains a significant amount of antibiotic resistant *E*. *coli*, specifically extended-spectrum beta-lactamase-producing *E*. *coli*^7^. Particularly, multidrug-resistant (MDR) clones (normally defined as those resistant to three or more drug classes^8^) are of great concern. Past studies have documented the presence of MDR *E*. *coli* isolates in wastewater on the basis of phenotypic resistance testing^9^, but a comprehensive analysis of the clonal composition of MDR *E*. *coli* in wastewater employing whole genome analysis is largely lacking. Therefore, the current information on the genomic diversity of antibiotic resistant *E*. *coli* in wastewater is very limited. Recent metagenomic studies have documented that human-associated bacteria are strongly reduced in the wastewater and its treatment process^10^. To investigate the genomic diversity as well as virulence genes and resistance determinants for wastewater *E*. *coli*, we proceeded as sketched in Fig. 1. We collected 1178 *E*. *coli* isolates from a waste treatment plant’s inflow and outflow in the city of Dresden, Germany. We selected 20 antibiotics, which are the most prescribed ones in the area from which the wastewater inflow originates (data provided by the public health insurer AOK). We analyzed the isolates’ resistance to these 20 antibiotics and selected 103 isolates for whole genome sequencing. Our analysis reveals a surprisingly high genomic diversity of MDR *E*. *coli* in the wastewater with very flexible genomes harboring a high variation of virulence genes and resistance determinants. Using this diversity, we developed a computational approach to identify not only known, but also novel genes correlating with resistance.

## Results

### The wastewater pan-genome

The concept of evolution implies that genomes of organisms of the same species differ. Differences range from single nucleotide polymorphisms to large genome rearrangements. As a consequence, *E*. *coli* possesses a core of genes present in all genomes, as well as genes only present in some genomes, or even just in one. The union of all of these genes is called the pan-genome. It is believed, that the *E*. *coli* core genome comprises around 1400–1500 genes, while the pan-genome may be of infinite size^11^.

To assess the degree of genomic flexibility of the wastewater isolates, we relate the wastewater pan-genome and the wastewater core genome. At 16582 genes, the wastewater pan-genome is nearly six times larger than the wastewater core genome of 2783 genes, a reservoir of some 14000 genes. Despite this large reservoir, the size difference of nearly 1000 genes between the wastewater *E*. *coli* core genome and the whole species core genome suggests that the full diversity of *E*. *coli* is still not covered in our wastewater sample.

The balance between maintaining the core genome and spending energy on acquisition of new genetic material can be captured by the ratio of the core genome size and the average genome size, which is 4700 genes in our sample. This means that only 1400/4700 = 30% of genes in our wastewater *E*. *coli* are core genes. Most of the non-core genes are very unique and appear only in one or two isolates each. More precisely, 50% of the pan-genome genes appear in only one or two isolates each. This implies that the investigated wastewater *E*. *coli* are highly individual.

**Figure 2:**
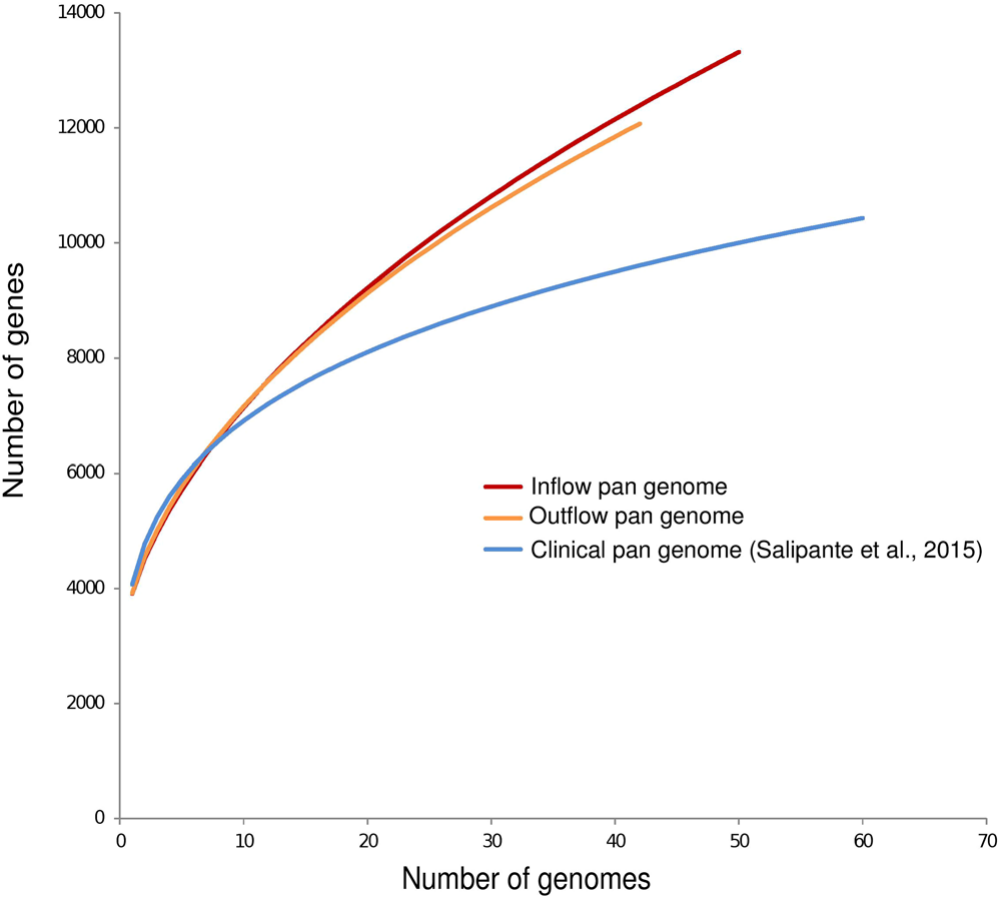
The pan-genome at the outflow has the same size as at the inflow, suggesting that highly flexible *Escherichia coli* emerge from a treatment plant. The wastewater pan-genome is larger than a clinical pan-genome and of similar size to (see Table 1) highly diverse samples comprising pathogenic, commensal, and lab *Escherichia coli*, as well as *Shigella*.

This high diversity is also illustrated in Fig. 2, which compares the wastewater *E*. *coli* to a clinical dataset of *E*. *coli*. The figure clearly shows that the *E*. *coli* of clinical origin are more homogeneous and hence their pan-genome is smaller. In contrast, the diversity of the wastewater *E*. *coli* match other datasets comprising mixtures of commensal and pathogenic *E*. *coli*, as well as *Shigella* genomes (see Table 1). This underlines the great diversity of *E*. *coli* genomes in the wastewater. Interestingly, the variation of the wastewater genomes after the treatment plant was not reduced.

**Table 1:** Highly diverse samples comprising pathogenic, commensal, and lab *Escherichia coli*, as well as *Shigella*.

Ref	Pan	Core	Strains	Path.	Comm.	Lab	*Shig*.
This study	16582	2783	92	28	62	0	0
Kaas *et al*.^54^	16373	1702	186	171			15
Vieira *et al*.^55^	14986	1957	29	21	8	0	6
Gordienko *et al*.^56^	12000	2000	32	16	6	3	7
Lukjancenko *et al*.^57^	13000	1472	53	35	11	7	0
Rasko *et al*.^58^	13000	2344	17	14	1	2	0
Touchon *et al*.^28^	11432	1976	20	10	3	0	7

Path. = Pathological.

Comm. = Commensal.

Lab. = Laboratory.

Shig. = *Shigella*.

### Resistance genes in the wastewater pan-genome

**Figure 3:**
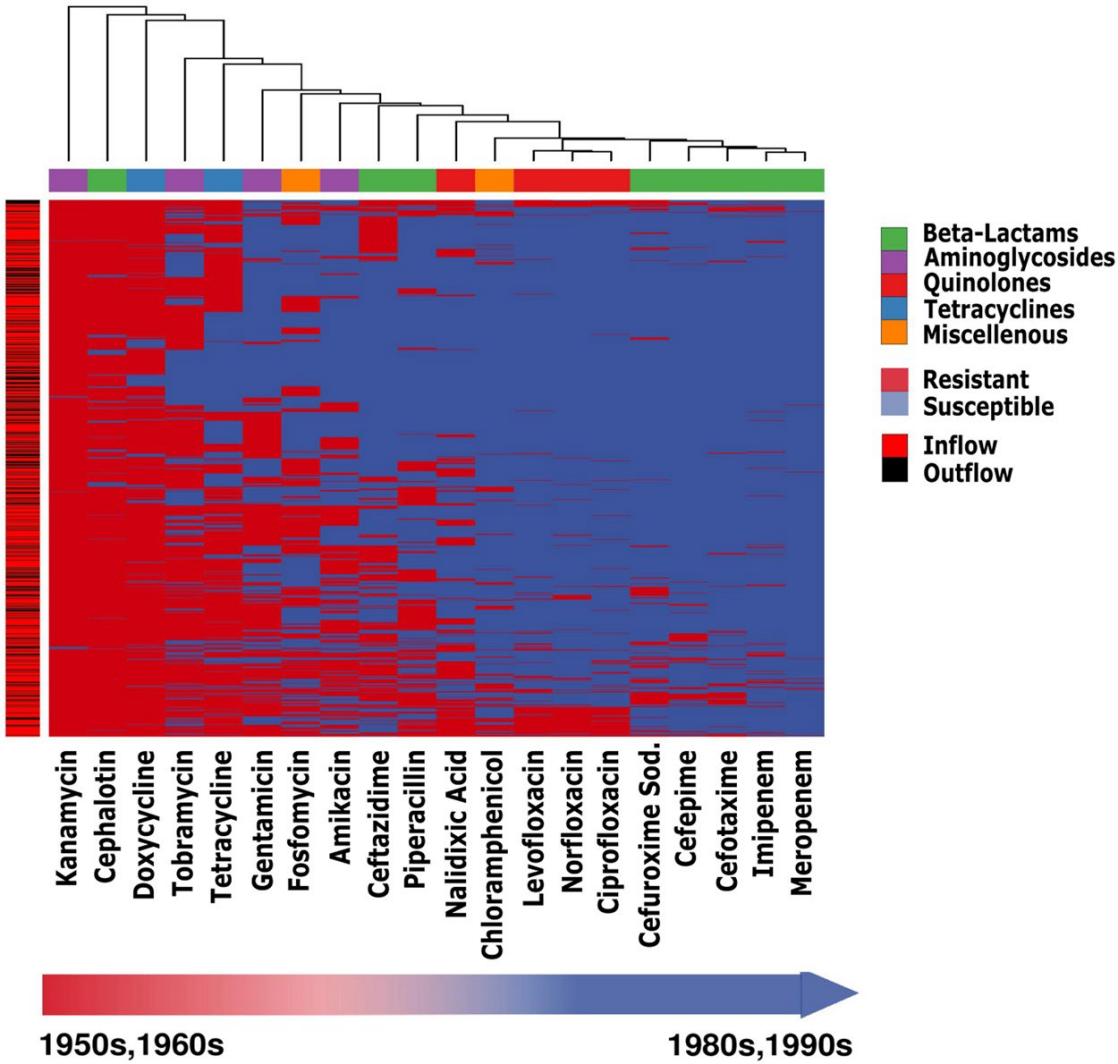
1178 Wastewater *Escherichia coli* isolates were tested for antibiotic resistance to 20 antibiotics covering 4 main classes as well as the Miscellenous class (chloramphenicol and fosfomycin). Nearly all isolates are multi-drug resistant. Isolates were highly susceptible to carbapenems (meropenem and imipenem) which are beta-lactams. Isolates were also more susceptible to fluoroquinolones than to tetracyclins and aminoglycosides. The outflow isolates (n = 322) show similar resistance as inflow (n = 856) (p-value 0.0001), suggesting that wastewater treatment is not reducing resistance development.

Wastewater *E*. *coli* are known to host antibiotic resistance genes. While there are many known resistance genes (see e.g. CARD^12^), they fall mostly into a few groups, such as beta-lactamases. Here, we seek to confirm and expand the space for resistance genes. Firstly, we measured antibiotic resistance in all 1178 isolates to the 20 antibiotics. As mentioned above, these 20 antibiotics include the most widely used antibiotics in the wastewater plant’s region. They included kanamycin and cephalotin, which are under debate regarding their intrinsic resistance, but to which *E*. *coli* are shown to be susceptible in many studies^13–18^. Figure 3 shows that 4 isolates are susceptible to kanamycin and 45 to cephalotin.

Figure 3 reveals a high degree of resistance and big differences between different antibiotics, including a general trend indicating greater resistance to antibiotics that have been available for longer. Specifically, isolates were significantly more resistant to antibiotics from the 50 s and 60 s namely, chloramphenicol, cephalotin, doxycycline, fosfomycin, gentamicin, kanamycin, nalidixic acid, tetracycline & tobramycin, than the more recent antibiotics (Welch test, p-value < 0.0025, also significant without including kanamycin and cephalotin). However, there is no significant difference in the number of resistances between isolates from the inflow and the outflow (p-value 0.0001), suggesting that wastewater treatment is not affecting resistance.

Next, we tried to predict the resistance observed in Fig. 3 using known resistance genes. To this end, we employed ResFinder^19^ and could predict resistances across all classes of drugs (see Supp Fig. 4) at an accuracy of 46%. While these are promising results, they show also that the known resistance genes used in the analysis are not sufficient for a perfect prediction. Therefore, we wanted to expand the link from genotype to phenotype by going beyond known resistance genes. Thus, we correlated the presence of each gene in the sequenced isolates with their phenotypic antibiotic resistance profiles.

**Figure 4:**
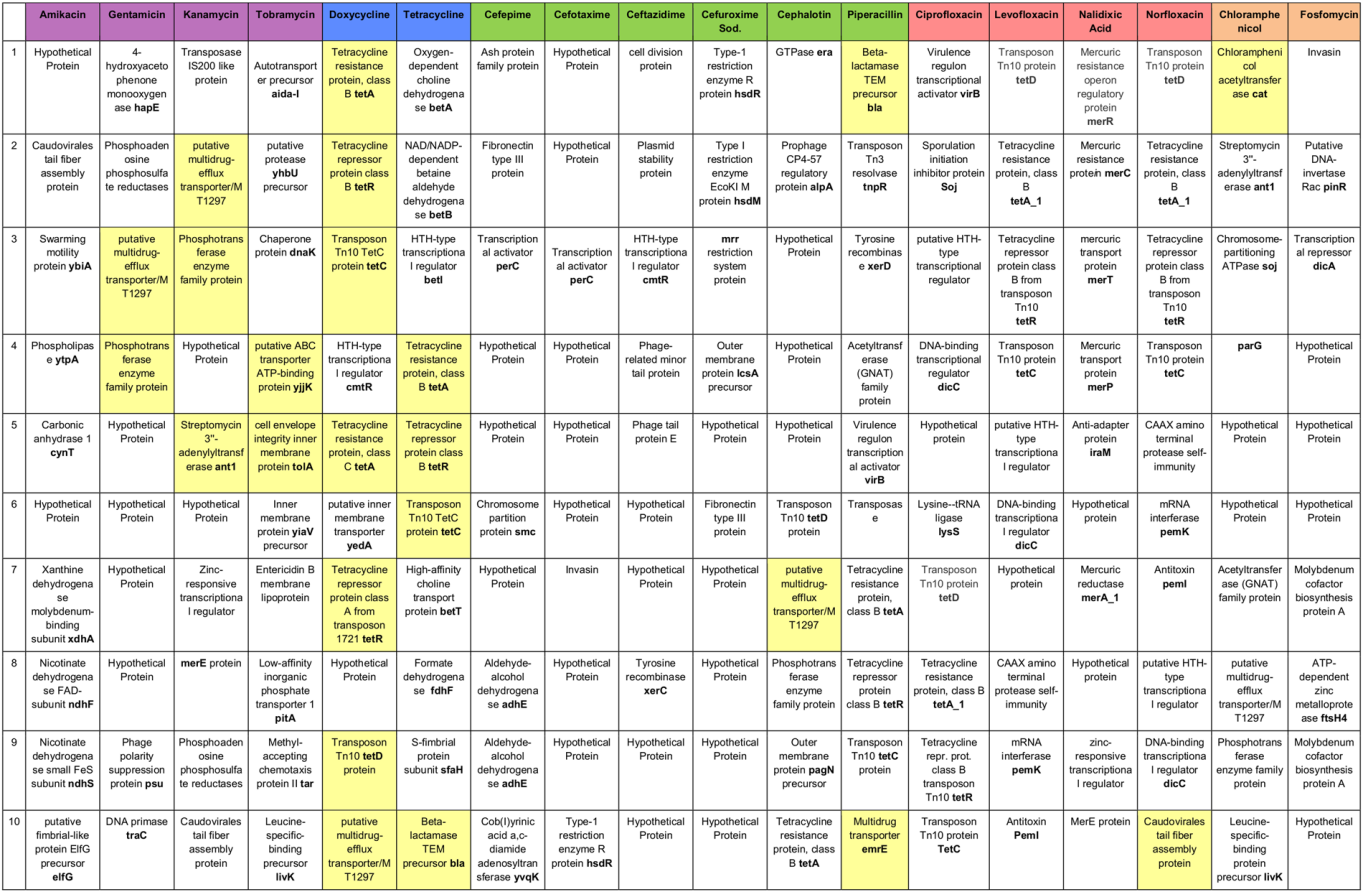
Top 10 correlating genes for 18 antibiotics from correlation of genomes to resistance phenotype. Antibiotics were color-coded based on antibiotic class following the scheme in Fig. 3. The highlighted yellow boxes represent genes involved in resistance to the respective antibiotics based on available literature.

Meropenem and imipenem are clinically important antibiotics, which are very effective, as can be seen in Fig. 3. Hardly any of the isolates are resistant to them. Since both drugs work so well, correlation of presence and absence of genes to resistance/susceptibility will be naturally poor. Hence, we have excluded both compounds from the correlation analysis. For each of the 18 remaining antibiotics, we list the top ten correlating genes in the table shown in Fig. 4. These 180 genes comprise 88 unique confirmed genes, including many well-known resistance genes, such as efflux pumps (MT1297 and *emr*E), membrane and transport proteins (*aida-*I, *yia*V, *yij*K, *pit*A, *ics*A, and *pag*N), tetracycline (*tet*A, *tet*R, and *tet*C), chloramphenicol (*cat*), and piperacillin (the beta lactamase *bla*2) resistance genes. Based on available literature, genes that are known to mediate resistance against the respective antibiotic (e.g. *tetA* mediates resistance against tetracycline and *cat* mediates resistance against chloramphenicol) were highlighted in yellow. However, the 180 genes also comprise a large number of open reading frames encoding hypothetical proteins (41) and genes not yet linked to antibiotic resistance (116). These genes have to be studied further to determine whether they are novel resistance genes or just correlating. (e.g. because they are on the same genetic element with a resistance gene). As a consequence, the *tet* gene, which is a known resistance gene against tetracyclins is highlighted in the table shown in Fig. 4, but occurrences of *tet*, which appear among the quinolones are not highlighted in yellow. Nearly all of the identified genes are found both in inflow and outflow genomes suggesting that the wastewater treatment does not impact on the presence or absence of known resistance genes and genes correlating with resistance.

### Virulence genes

**Figure 5:**
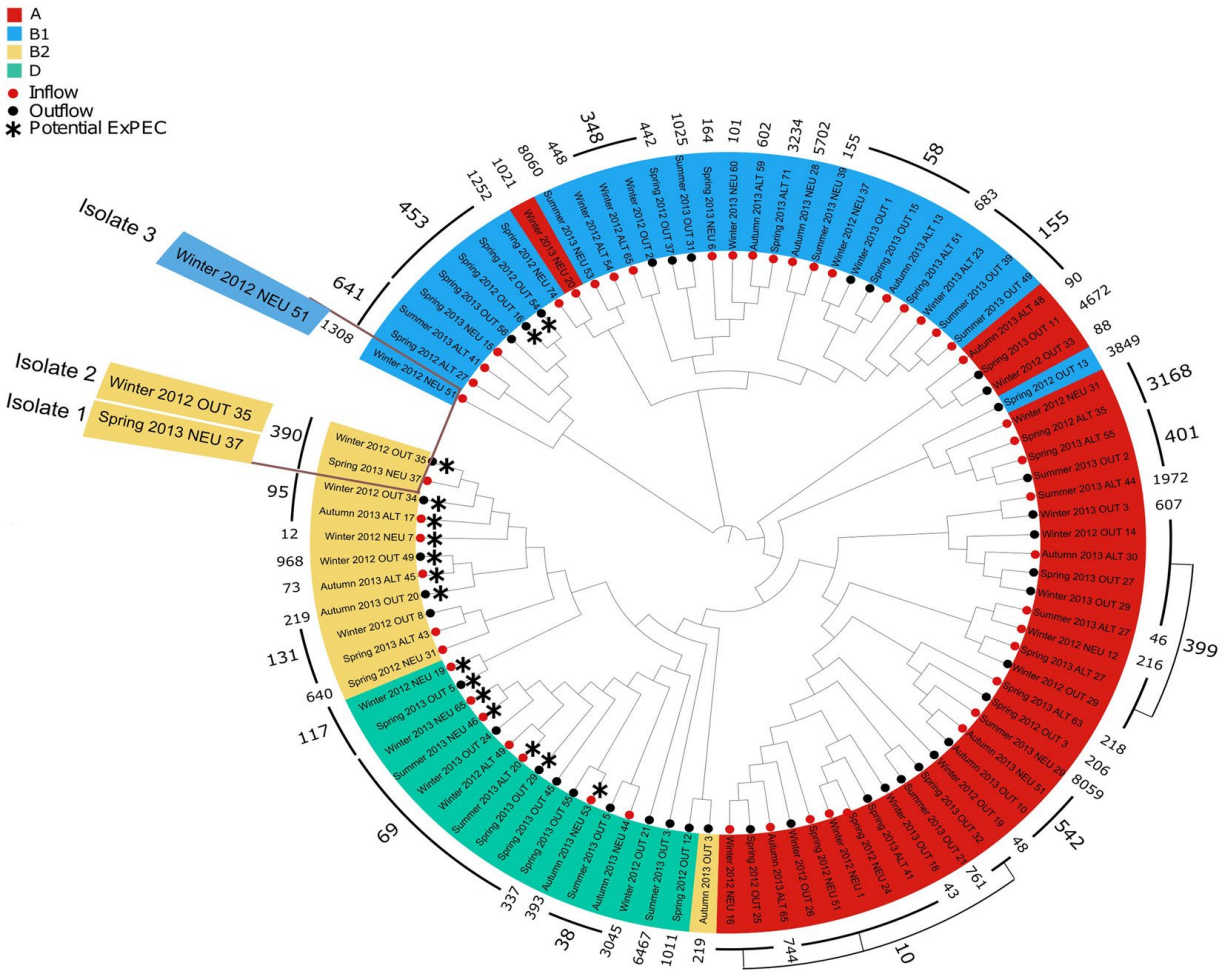
Phylogeny of wastewater *Escherichia coli* from the inflow (n = 50) and the outflow (n = 42) of a wastewater treatment plant. Phylogenetic tree, multi-locus sequence types (shown as numbers in black), and phylogroups of 92 sequenced wastewater *Escherichia coli* isolates reveal 16 potential ExPEC isolates (marked with a black star) in phylogroups B2 (yellow) and D (green), which are associated with pathogenicity. Half of these 16 isolates stem from the outflow of the treatment plant.

Generally, *E*. *coli* strains exhibit great variation. Many exist as harmless commensals in the human gut, but some are classified as intra- (InPEC) or extra-intestinal pathogenic *E*. *coli* (ExPEC^20^). Based on their virulence genes profile the pathogenic potential of *E*. *coli* isolates can be determined^7^. The sequenced isolates contain some 700 of nearly 850 *E*. *coli* protein sequences representing 400 virulence factors and their isoforms in the virulence factor database^21^, averaging to 153 and to 155 virulence factors per isolate for inflow and outflow, respectively. Hence, there is no significant difference (Welch test, CI 95%) between inflow and outflow. In particular, we found combinations of virulence factors for 16 isolates (see methods), which are indicative of ExPEC. Eight of these 16 isolates were obtained from the outflow of the treatment plant (see Fig. 5).

Besides the presence of known virulence factors, pathogenic isolates are more likely to be member of certain multi-locus sequence types^22^ and phylogroups^23,24^. Broadly, *E*. *coli* has seven phylogroups, A, B1, B2, D D, E, F^25^. Commensal as well as intestinal pathogenic *E*. *coli* fall mostly into groups A and B1^26^ and ExPEC into B2 and D^23^. Figure 5 shows a phylogenetic tree of the sequenced wastewater *E*. *coli* isolates along with the commensal phylogroups A (red) and B1 (blue) and the pathogenicity-associated groups B2 (yellow) and D (green), as well as the finer-grained multi-locus sequence types. The tree is based on genomic variations compared to the reference genome of *E*. *coli* K12 MG1655. Figure 5 reveals that nearly one third of isolates belong to group B2 and D, in which ExPEC are usually found. In particular, B2 and D include 14 of the 16 potential ExPEC isolates. Remarkably, half of the B2 and D isolates are from the wastewater treatment plant’s outflow. To provide a scale for phylogenetic relationships of isolates in the tree, consider isolates 1, 2 and 3; isolate 1 is very close to isolate 2, but very far from 3. Isolates 1 and 2 have 25,218 single nucleotide polymorphisms (SNPs) in common, while 1 and 3 share only 4,928 SNPs. Overall, the number of shared SNPs ranges from 647 to 25218 averaging at 5271 SNPs (at a standard deviation of 3514).

## Discussion

### Pan and core genome

It is well known that wastewater treatment reduces the bacterial abundance, in addition a recent metagenomic study has shown that the bacterial community in wastewater is very different to the human gut community and that the number of detected genera is reduced in the wastewater^10^. Consequently, our expectation was that the genomic diversity of *E*. *coli* should be reduced. We were very surprised to find an unexpectedly high genomic diversity, which is illustrated in the large pangenome. A possible explanation for this high genomic diversity is that the *E*. *coli* cells within the wastewater originate not only from human faeces, but also from a multitude of different animal faeces collected via the surface runoff into the sewers. This would also explain why the pangenome of the wastewater *E*. *coli* is considerably larger than the clinical pangenome reported by Land *et al*.^27^. Generally, many authors have pointed out that *E*. *coli* has a large and flexible pan genome. Lapierre *et al*. argue that *E*. *coli* appears to have unlimited ability to absorb genetic material and hence its pan genome is open^11^. In a recent study comprising over 2000 genomes Land *et al*. put this into numbers and arrive at a pan genome of 60000–89000 gene families for over 2000 sequenced *E*. *coli* genomes^27^. The study by Land *et al*. (24) is based on clinical isolates, in contrast our study is the first, which has calculated the pangenome of *E*. *coli* for wastewater. Interestingly, our results seem to be in concordance and suggest that within our study we still have not reached the saturation of the detected diversity (Fig. 2), indicating that the full genomic diversity of *E*. *coli* in the wastewater is probably even larger than what we report here. Worryingly, this is also reflected in a high diversity of resistance and virulence genes. This documents that the wastewater contains a significant amount of multi-drug resistant (MDR) *E*. *coli*, which also carry a suit of virulence genes suggesting that some of those MDR have a pathogenic potential. Furthermore, we did not find a significant difference in genomic diversity between inflow and outflow of the wastewater treatment plant, suggesting that selection against genome diversity and resistance determinants does not seem to occur.

### Pathogenic potential and resistance

Resistant bacteria may or may not be pathogenic. While ultimate proof for pathogenicity can only be obtained from *in vivo* studies, we wanted to analyse the genomes for markers likely to be found in pathogenic bacteria. Here we chose to consider three independent approaches: classification by phylogenetic groups, by multi-locus sequence tags, and by identification of specific virulence factors (see methods). While the three approaches showed consistent results, they are by no means proof for pathogenicity, since there can be exceptions to these classification rules. As an example, consider the strain ED1a (O81), which was isolated from a healthy man, but belongs to the phylogenetic group B2^28^. Similarly, pathogenicity may not only arise from the acquisition of genes, but also from the loss^29^.

Regarding resistance there are similar confounding factors. Bacteria may be inherently resistant since generally antibiotic resistance is ancient^30^ and naturally occurring in the environment. Nonetheless, there are pronounced differences between pristine and human environments^24^. This is also supported by Fig. 3, which shows that antibiotics introduced in the 50 s and 60 s have more resistances than those introduced later (p-value < 0.0025), which suggests, that the naturally occurring resistances do not play a major role in the emergence of observed resistances.

### From clinic to river

We have shown that there are *E*. *coli* at the wastewater outflow, which are multi-drug resistant and have markers found in pathogenic bacteria. But are they abundant enough to have an impact in the aquatic system they are released into? They do. The percentage of possibly pathogenic *E*. *coli* in the outflow is considerable and may correspond to a large absolute amount. Caucci *et al*. quantified the amounts of eight antibiotic resistance genes at the inflow and outflow of a wastewater treatment plant^31^. They found in the order of 10^4^ to 10^8^ copies at the inflow and a reduction of three orders of magnitude at the outflow. They argue that the reduction relates directly to the bacterial removal rate of the treatment plant and their numbers show that despite removal, substantial amounts of resistance genes remain. Also, the number of *E*. *coli* are assessed at the wastewater treatment plant regularly and they are between 10^4^ and 10^5^ Colony Forming Units (CFU)/ml for the inflow and 10^2^ and 10^3^ (CFU/ml) for the outflow respectively and the numbers are comparable to other studies^32^. Generally, if an average of 100 *E*. *coli* colony forming units (CFU) are released per ml, then 10^13^ CFUs per day are released (assuming a release of 10^5^ m^3^ per day). This is in accordance with Manaia *et al*., who showed that 10^10^–10^14^ CFU of ciprofloxacin-resistant bacteria are released by a mid-sized wastewater treatment plant^33^. Supporting these results, a study in a Japanese river shows the presence of pathogenic *E*. *coli*^34^. In this study they sequenced over 500 samples from the Yamato river and most of their prevalent multi-drug resistant and clinical strains are also present in our samples. In a related study, Czekalski *et al*. found that particle-associated wastewater bacteria are the responsible source for antibiotic resistance genes in the sediments of lake Geneva in Switzerland^35^. Assuming that the river Elbe is comparable to these aquatic systems, it suggests, that the urban environment (including clinics) and river are connected with wastewater treatment plants in between.

### Composition of phylogroups

It is interesting to compare the breakdown into phylogenetic groups of wastewater *E*. *coli* to compare samples from human and animal environments. It is, e.g., known that the phylogenetic group B2 is more abundant among commensal *E*. *coli* from human faeces (43%) than from farm animals (11%)^36^. Therefore, the composition of wastewater *E*. *coli* as shown in Fig. 5 resembles commensal *E*. *coli* from farm animals more closely. Similarly, Tenaillon *et al*. find that groups A and B1 make up one third in human faeces^36^, whereas we find two thirds. This suggests that animal feces play an important role for resistance also of urban wastewater treatment plants. Besides the diverse environments such as soils and activated sludge, animal feces are probably part of the explanation for the high observed genomic diversity.

### Random sampling and novel resistance mechanisms

The initial 1178 isolates were sampled randomly over different times of the year, from two different inflows and the outflow of the wastewater treatment plant. In contrast, the 103 sequenced isolates were chosen in such way that all of the phenotypes encountered were represented (see methods). Within a phenotype group isolates were chosen randomly. This random, but representative choice and the subsequent link from genotype to phenotype is an example of high-throughput hypothesis-free analysis. And although, there was no pre-defined resistance mechanism, which we aimed to hit, many of the well-known resistance genes were ranked high. This supports the hope that high-throughput, hypothesis-free methods such as deep sequencing will help to uncover novel resistance mechanisms and in particular that some of the top correlating genes will prove to have a causal link to resistance. The results show that the here outlined computational approach to correlate genomic and phenotypic information for wastewater *E*. *coli* significantly assists to identify a larger part of the existing resistome of *E*. *coli*. However, a limitation to the method is that it can pinpoint correlating genes if resistances have manifested themselves, but not when they are yet to emerge. For future investigations, it will be interesting to expand the analysis to mutations within genes (e.g. there are well-known mutations in gyrA and parC conferring quinolone resistance) and in non-coding regions (mutations in the promoter region of ampC conferring beta-lactam resistance).

## Conclusion

Overall, we have shown for the first time that *E*. *coli* isolates from wastewater have a surprisingly large pan-genome, which harbors virulence genes, known resistance genes and genes correlating with resistance. We developed a computational approach based on genomic and phenotypic correlation for *E*. *coli* and show that applying this to wastewater will discover novel parts of the resistome in *E*. *coli*. Finally, together with the estimates on absolute *E*. *coli* abundance, we could demonstrate that there is a considerable pathogenic potential in the outflow of a wastewater treatment plant. Using *E*. *coli* as an example, this study demonstrates the importance of investigating wastewater with modern bioinformatics and strain specific genomic analysis in order to estimate the extent of genomic variation and resistance determinants for bacteria with clinical relevance present in the environment.

## Methods

### Collection

1178 samples were collected from the municipal wastewater treatment plant Dresden, Germany. Samples were collected on 11/4/2012 (Spring 2012), 30/7/2012 (Summer 2012), 21/1/2013 (Winter 2012), 27/3/2013 (Spring 2013), 6/8/2013 (Summer 2013), 14/10/2013 (Autumn 2013), and 17/12/2013 (Winter 2013). Samples were collected either at the outflow (n = 322, OUT) or at one of two inflow locations (n = 856, Altstadt ALT and Neutstadt NEU), representing the area south and north of the river Elbe.

### Isolation

*E*. *coli* and total coliforms bacteria were enumerated via serial fold dilution plating of the original wastewater (triplicate samples). Wastewaters were diluted in double distilled water, until the enumeration of bacterial colonies was possible. *E*. *coli* and coliform counts were always performed in triplicates. The *E*. *coli* colonies were selected and picked after overnight growth at 37 °C on a selective chromogenic media (OXOID Brilliance *Escherichia coli*/Coliform Selective Agar, Basingstoke, England). All single colonies recognised as chromogenically positive E.coli were picked. To reduce the dilution effect on *E*. *coli* diversity, extra effort was placed for colony picking at the lowest fold dilution. To minimize the risk of colony contamination, picked colonies were spiked a second time on the same selective media and pure single colonies were grown overnight on LB media at 37 °C and stored on glycerol stock at −80 °C. For the cell counting we used mFC Agar and incubated the plates at 44 °C for 20 h ( ± 2 h).

### Resistance phenotyping

Antibiotic resistance phenotypes were determined by the agar diffusion method using 20 antibiotic discs (OXOID, England) according to EUCAST (or CLSI when EUCAST was not available)^7,9^. The selected drugs belong to the most commonly prescribed antibiotics for diseases caused by bacteria according to the German health insurance AOK Plus: piperacillin (100 *µg*), nalidixic acid (30 *µg*), chloramphenicol (30 *µg*), imipenem (10 *µg*), cefotaxime (30 *µg*), cephalotin (30 *µg*), kanamycin (30 *µg*), tetracycline (30 *µg*), gentamicin (10 *µg*), amikacin (30 *µg*), ciprofloxacin (5 *µg*), fosfomycin (50 *µg*), doxycycline (30 *µg*), cefepime (30 *µg*), ceftazidime (10 *µg*), levofloxacin (5 *µg*), meropenem (10 *µg*), norfloxacin (10 *µg*), cefuroxime sod. (30 *µg*), tobramycin (10 *µg*)^31^. After 24 hours of incubation at 37 °C, the resistance diameters were measured. Clustering of antibiotics and of isolates was performed using the R function heatmap.2 from the R library^37^ Heatplus and hierarchical clustering of matrices based on Euclidean distances between isolates and between antibiotics.

### Sequencing

To select isolates representative of phenotype, we clustered isolates according to the diameters of inhibition zone against the 20 antibiotics using k-means clustering based on Euclidean distances between isolates (vectors of 20 inhibition zone diameters). The analysis and graphs were produced using R version 3.2.4^37^. As clusters may be highly skewed in number of cluster members, we tested all cluster numbers from 1 to 100 and plotted within class sum of squares against *k*. At *k* = 47, the sum of squares tails off and there is a steep local decrease, so that *k* = 47 was fixed as k-means parameter. We obtained 103 isolates, which were subsequently used for sequencing and further analysis. To further validate the choice, we plotted the average number of resistances against number of isolates and antibiotics vs. number of isolates for the total 1178 and the selected 103 isolates (see Supp Fig. 1) and concluded that both distributions are roughly similar. 3000 ng DNA were extracted from each of the 103 selected isolates using MasterPure extraction kit (Epicentre) according to the manufacturer’s instructions. Sequencing was performed on an Illumina MiSeq system using V3 chemistry and the Nextera XT kit for library preparation.

### Assembly

Genomes were assembled with Abyss (version 1.5.2)^38^. In order to optimize *k* for the best assembly, k-mer values had to be empirically selected from the range of 20–48 (see Supp Fig. 2) on a per sample basis to maximize contiguity^3^. To determine the k-mer length that achieved highest contiguity, the 28 assemblies per draft genome/isolate were compared based on *N50* values. 11 assemblies with an *N50* statistic of less than 5 × 10^4^ bp were excluded^39^.

### Genes

Reference gene clusters were computed from 58 complete *E*. *coli* genomes (see Table 2) available in June 2015 from NCBI. Genes were identified in wastewater and reference genomes using Prokka (version 1.11)^40^. Genes were clustered at 80% using CD-HIT^41^ (version 4.6.3, arguments -n 4 -c 0.8 -G 1 -aL 0.8 –aS 0.8 -B 1). Genes with over 90% sequence identity, but only 30% coverage, as well as genes with 80% or greater identity and covered to phage and virus sequences^42^ were discarded. A gene cluster is defined to be present in an isolate if there is a Prokka gene in the genome, which is longer than 100 amino acids and has over 80% sequence identity and coverage against the gene cluster representative.

**Table 2:** Accession numbers of 92 de novo assembled wastewater *Escherichia coli* genomes.

Bioproject	Biosample	Accession	strain
PRJNA380388	SAMN06641941	NBBP00000000	Escherichia coli Win2013_WWKa_OUT_3
PRJNA380388	SAMN06641940	NBBQ00000000	Escherichia coli Win2013_WWKa_OUT_29
PRJNA380388	SAMN06641933	NBBR00000000	Escherichia coli Win2013_WWKa_OUT_18
PRJNA380388	SAMN06641932	NBBS00000000	Escherichia coli Win2013_WWKa_OUT_24
PRJNA380388	SAMN06641931	NBBT00000000	Escherichia coli Win2013_WWKa_OUT_1
PRJNA380388	SAMN06641928	NBBU00000000	Escherichia coli Win2013_WWKa_NEU_65
PRJNA380388	SAMN06641927	NBBV00000000	Escherichia coli Win2013_WWKa_NEU_20
PRJNA380388	SAMN06641926	NBBW00000000	Escherichia coli Win2013_WWKa_NEU_60
PRJNA380388	SAMN06641901	NBBX00000000	Escherichia coli Win2013_WWKa_ALT_23
PRJNA380388	SAMN06641884	NBBY00000000	Escherichia coli Win2012_WWKa_OUT_49
PRJNA380388	SAMN06641883	NBBZ00000000	Escherichia coli Win2012_WWKa_OUT_8
PRJNA380388	SAMN06641882	NBCA00000000	Escherichia coli Win2012_WWKa_OUT_34
PRJNA380388	SAMN06641881	NBCB00000000	Escherichia coli Win2012_WWKa_OUT_35
PRJNA380388	SAMN06641880	NBCC00000000	Escherichia coli Win2012_WWKa_OUT_29
PRJNA380388	SAMN06641879	NBCD00000000	Escherichia coli Win2012_WWKa_OUT_26
PRJNA380388	SAMN06641878	NBCE00000000	Escherichia coli Win2012_WWKa_OUT_33
PRJNA380388	SAMN06641877	NBCF00000000	Escherichia coli Win2012_WWKa_OUT_21
PRJNA380388	SAMN06641876	NBCG00000000	Escherichia coli Win2012_WWKa_OUT_2
PRJNA380388	SAMN06641875	NBCH00000000	Escherichia coli Win2012_WWKa_NEU_7
PRJNA380388	SAMN06641874	NBCI00000000	Escherichia coli Win2012_WWKa_OUT_14
PRJNA380388	SAMN06641873	NBCJ00000000	Escherichia coli Win2012_WWKa_NEU_51
PRJNA380388	SAMN06641872	NBCK00000000	Escherichia coli Win2012_WWKa_NEU_31
PRJNA380388	SAMN06641871	NBCQ00000000	Escherichia coli Win2012_WWKa_NEU_37
PRJNA380388	SAMN06641870	NBCR00000000	Escherichia coli Win2012_WWKa_NEU_16
PRJNA380388	SAMN06641869	NBCS00000000	Escherichia coli Win2012_WWKa_NEU_19
PRJNA380388	SAMN06641868	NBCT00000000	Escherichia coli Win2012_WWKa_NEU_12
PRJNA380388	SAMN06641867	NBCU00000000	Escherichia coli Win2012_WWKa_ALT_65
PRJNA380388	SAMN06641866	NBCV00000000	Escherichia coli Win2012_WWKa_NEU_1
PRJNA380388	SAMN06641865	NBCW00000000	Escherichia coli Win2012_WWKa_ALT_49
PRJNA380388	SAMN06641864	NBCX00000000	Escherichia coli Win2012_WWKa_ALT_54
PRJNA380388	SAMN06641863	NBCY00000000	Escherichia coli Sum2013_WWKa_OUT_5
PRJNA380388	SAMN06641862	NBCZ00000000	Escherichia coli Sum2013_WWKa_OUT_39
PRJNA380388	SAMN06641861	NBDA00000000	Escherichia coli Sum2013_WWKa_OUT_49
PRJNA380388	SAMN06641860	NBDB00000000	Escherichia coli Sum2013_WWKa_OUT_3
PRJNA380388	SAMN06641859	NBDC00000000	Escherichia coli Sum2013_WWKa_OUT_31
PRJNA380388	SAMN06641858	NBDD00000000	Escherichia coli Sum2013_WWKa_OUT_2
PRJNA380388	SAMN06641857	NBDE00000000	Escherichia coli Sum2013_WWKa_OUT_21
PRJNA380388	SAMN06641856	NBDF00000000	Escherichia coli Sum2013_WWKa_NEU_53
PRJNA380388	SAMN06641855	NBDG00000000	Escherichia coli Sum2013_WWKa_NEU_46
PRJNA380388	SAMN06641854	NBDH00000000	Escherichia coli Sum2013_WWKa_NEU_39
PRJNA380388	SAMN06641853	NBDI00000000	Escherichia coli Sum2013_WWKa_ALT_44
PRJNA380388	SAMN06641852	NBDJ00000000	Escherichia coli Sum2013_WWKa_NEU_29
PRJNA380388	SAMN06641851	NBDK00000000	Escherichia coli Spr2013_WWKa_OUT_27
PRJNA380388	SAMN06641844	NBDL00000000	Escherichia coli Sum2013_WWKa_ALT_41
PRJNA380388	SAMN06641843	NBDM00000000	Escherichia coli Sum2013_WWKa_ALT_27
PRJNA380388	SAMN06641842	NBDN00000000	Escherichia coli Spr2013_WWKa_OUT_56
PRJNA380388	SAMN06641841	NBDO00000000	Escherichia coli Sum2013_WWKa_ALT_20
PRJNA380388	SAMN06641840	NBJM00000000	Escherichia coli Spr2013_WWKa_OUT_5
PRJNA380388	SAMN06641839	NBJN00000000	Escherichia coli Spr2013_WWKa_OUT_55
PRJNA380388	SAMN06641838	NBJO00000000	Escherichia coli Spr2013_WWKa_OUT_32
PRJNA380388	SAMN06641837	NBJP00000000	Escherichia coli Spr2013_WWKa_OUT_45
PRJNA380388	SAMN06641822	NBJQ00000000	Escherichia coli Spr2013_WWKa_OUT_15
PRJNA380388	SAMN06641821	NBJR00000000	Escherichia coli Spr2013_WWKa_OUT_29
PRJNA380388	SAMN06641820	NBJS00000000	Escherichia coli Spr2013_WWKa_NEU_6
PRJNA380388	SAMN06641819	NBJT00000000	Escherichia coli Spr2013_WWKa_OUT_11
PRJNA380388	SAMN06641818	NBJU00000000	Escherichia coli Spr2013_WWKa_NEU_15
PRJNA380388	SAMN06641817	NBJV00000000	Escherichia coli Spr2013_WWKa_NEU_37
PRJNA380388	SAMN06641816	NBJW00000000	Escherichia coli Spr2013_WWKa_ALT_63
PRJNA380388	SAMN06641815	NBJX00000000	Escherichia coli Spr2013_WWKa_ALT_71
PRJNA380388	SAMN06641814	NBJY00000000	Escherichia coli Spr2013_WWKa_ALT_51
PRJNA380388	SAMN06641813	NBJZ00000000	Escherichia coli Spr2013_WWKa_ALT_55
PRJNA380388	SAMN06641812	NBKA00000000	Escherichia coli Spr2013_WWKa_ALT_43
PRJNA380388	SAMN06641811	NBKB00000000	Escherichia coli Spr2013_WWKa_ALT_27
PRJNA380388	SAMN06641810	NBKC00000000	Escherichia coli Spr2013_WWKa_ALT_41
PRJNA380388	SAMN06641809	NBKD00000000	Escherichia coli Spr2012_WWKa_OUT_37
PRJNA380388	SAMN06641808	NBKE00000000	Escherichia coli Spr2012_WWKa_OUT_54
PRJNA380388	SAMN06641807	NBKF00000000	Escherichia coli Spr2012_WWKa_OUT_25
PRJNA380388	SAMN06641806	NBKG00000000	Escherichia coli Spr2012_WWKa_OUT_3
PRJNA380388	SAMN06641805	NBKH00000000	Escherichia coli Spr2012_WWKa_OUT_16
PRJNA380388	SAMN06641804	NBKI00000000	Escherichia coli Spr2012_WWKa_OUT_13
PRJNA380388	SAMN06641803	NBKJ00000000	Escherichia coli Spr2012_WWKa_NEU_74
PRJNA380388	SAMN06641802	NBKK00000000	Escherichia coli Spr2012_WWKa_OUT_12
PRJNA380388	SAMN06641801	NBKL00000000	Escherichia coli Spr2012_WWKa_NEU_31
PRJNA380388	SAMN06641800	NBKM00000000	Escherichia coli Spr2012_WWKa_NEU_51
PRJNA380388	SAMN06641799	NBKN00000000	Escherichia coli Spr2012_WWKa_NEU_24
PRJNA380388	SAMN06641798	NBKO00000000	Escherichia coli Spr2012_WWKa_ALT_27
PRJNA380388	SAMN06641797	NBKP00000000	Escherichia coli Spr2012_WWKa_ALT_35
PRJNA380388	SAMN06641796	NBKQ00000000	Escherichia coli Aut2013_WWKa_OUT_3
PRJNA380388	SAMN06641793	NBKR00000000	Escherichia coli Aut2013_WWKa_OUT_10
PRJNA380388	SAMN06641792	NBKS00000000	Escherichia coli Aut2013_WWKa_OUT_20
PRJNA380388	SAMN06641791	NBKT00000000	Escherichia coli Aut2013_WWKa_NEU_51
PRJNA380388	SAMN06641789	NBKU00000000	Escherichia coli Aut2013_WWKa_NEU_53
PRJNA380388	SAMN06641788	NBKV00000000	Escherichia coli Aut2013_WWKa_NEU_44
PRJNA380388	SAMN06641786	NBKW00000000	Escherichia coli Aut2013_WWKa_ALT_65
PRJNA380388	SAMN06641785	NBKX00000000	Escherichia coli Aut2013_WWKa_NEU_28
PRJNA380388	SAMN06641784	NBKY00000000	Escherichia coli Aut2013_WWKa_ALT_59
PRJNA380388	SAMN06641782	NBKZ00000000	Escherichia coli Aut2013_WWKa_ALT_48
PRJNA380388	SAMN06641780	NBLA00000000	Escherichia coli Aut2013_WWKa_ALT_45
PRJNA380388	SAMN06641779	NBLB00000000	Escherichia coli Aut2013_WWKa_ALT_30
PRJNA380388	SAMN06641778	NBLC00000000	Escherichia coli Aut2013_WWKa_ALT_17
PRJNA380388	SAMN06641777	NBLD00000000	Escherichia coli Aut2013_WWKa_ALT_13
PRJNA380388	SAMN06670745	NBNO00000000	Escherichia coli Win2012_WWKa_OUT_19

### Pan- and core-genome

To generate the pan- and core-genome size graph we followed the procedure in^3,28^. We had 92 genomes available. We varied *i* from one to 92. At each subset size *i*, we randomly selected *i* genomes and computed the sizes of the union (pan) and intersection (core) of gene clusters. This random selection was carried out 2000 times in each step.

### Gene clusters to rank genes by correlation to phenotype

Prokka genes were identified in all isolate genomes and then clustered with CD-HIT at 60% sequence identity and 50% coverage (arguments -n 4 -c 0.6 -G 1 -aL 0.8 -aS 0.5 -B 1). A 80% identity cutoff was also tried but dismissed, because the 60% threshold yielded 25% less clusters while adequately clustering homologous gene sequences with lower sequence similarity. This threshold value is also supported by the widespread default use of the BLOSUM62 matrix, the basis of which is sequences clustered by 62% sequence identity.

### Tree

The phylogenetic tree of 92 isolates was built following the procedure of^43,44^ using FastTree version 2.1^45^. Sequence reads were aligned to *E*. *coli* K12 MG 1665 and single nucleotide variant calling was carried out using GATK^46^. Quality control for variant calling was performed; variants supported by more than ten reads or likelihood score greater than 200 were always in the range of 84–99% of variants called per isolate with the exception of 2 isolates where only 59% and 60% of the variants were above the threshold for quality and supporting reads. FastTree 2.1^45^ was then used to build the maximum likelihood tree based on core single nucleotide polymorphisms derived from variant calling. **Phylogrouping**. For phylogrouping, the in-silico classification method established by Salipante *et al*.^3^ based on the classical classification by Clermont *et al*.^23^ was employed. BLAST was performed to check each genome assembly for presence or absence of the genetic elements *chuA* and *yjaA* and the DNA fragment TspE4.C2 with an identity cutoff ≥90%.

### MLST

Concerning epidemiology and Multi-Locus Sequence Typing, we used the webserver at https://cge.cbs.dtu.dk/services/MLST/ that follows the MLST scheme in^47^ for predicting MLSTs from whole genome sequence data^48^. 92 Draft genome assemblies were submitted and results were obtained; 2 isolates were unidentified demonstrating novel sequence types and have been assigned sequence types ST-8059 and ST-8060 by EnteroBase (https://enterobase.warwick.ac.uk/).

### Virulence factors

Virulence factors protein sequences were downloaded from VFDB: Virulence Factors database^21,49^. 2180 sequences, which are *E*. *coli* related, were chosen. Sequences were then clustered at 80% sequence identity using CD-HIT (version 4.6.3, arguments -n 4 -c 0.8 -G 1 -aL 0.8 -aS 0.8 –B 1) to 844 clusters. A virulence factor was considered present in an isolate’s genome if there is a Prokka gene in the genome that has over 80% sequence identity and coverage against the virulence factor cluster representative.

### ExPEC classification

There are intra- and extra-intestinal pathogenic *Escherichia coli*, which can be classified from the presence of virulence factors^50–53^. InPEC are characterised by the virulence factors stx1, stx2, escV, and bfpB. They are ExPEC if they contain over 20 of 58 virulence factors afa/draBC, bmaE, gafD, iha cds, mat, papEF, papGII, III, sfa/foc, etsB, etsC, sitD ep, sitD ch, cvaC MPIII, colV MPIX, eitA, eitC, iss, neuC, kpsMTII, ompA, ompT, traT, hlyF, GimB, malX, puvA, yqi, stx1, stx2, escV, bfp, feob, aatA, csgA, fimC, focG, nfaE, papAH, papC, sfaS, tsh, chuA, fyuA, ireA, iroN, irp2, iucD, iutA, sitA, astA, cnf1, sat, vat, hlyA, hlyC, ibeA, tia, and pic.

### Data availability

Genome assemblies of the analyzed isolates that support the findings of the study will be made available on the NCBI upon paper publication (see Table 2).

## Electronic supplementary material

Supplementary information
