# Supplementary material for: High genomic diversity of multi-drug resistant wastewater Escherichia coli
Source: Sci Rep. 2018 Jun 12;8:8928. doi: 10.1038/s41598-018-27292-6 (PMC5997705; doi:10.1038/s41598-018-27292-6)
Supplement: Supplementary file 1 — Supplementary information [file 41598_2018_27292_MOESM1_ESM.pdf]

High genomic diversity of multi-drug resistant wastewater *Escherichia coli*

Norhan Mahfouz[1,*], Serena Caucci[2,3,*], Eric Achatz[1], Torsten Semmler[4], Sebastian Guenther[4,5], Thomas U. Berendonk[2,*], and Michael Schroeder[1,*,#]

[1] Biotec, TU Dresden

[2] Institute for Hydrobiology, TU Dresden

[3] United Nations University Institute for Integrated Management of Material Fluxes and of Resources

[4] Institute of Microbiology und Epizootics, FU Berlin

[5] Institut für Pharmazie Pharmazeutische Biologie, Ernst-Moritz-Arndt-Universität Greifswald

* These authors contributed equally

# Correspondence: Michael Schroeder, ms@biotec.tu-dresden.de

Keywords: Antibiotic Resistance, Wastewater Treatment, Pan-Core genome, Environment

Conflict of interest statement: The authors declare no conflict of interest.

Supplementary Material

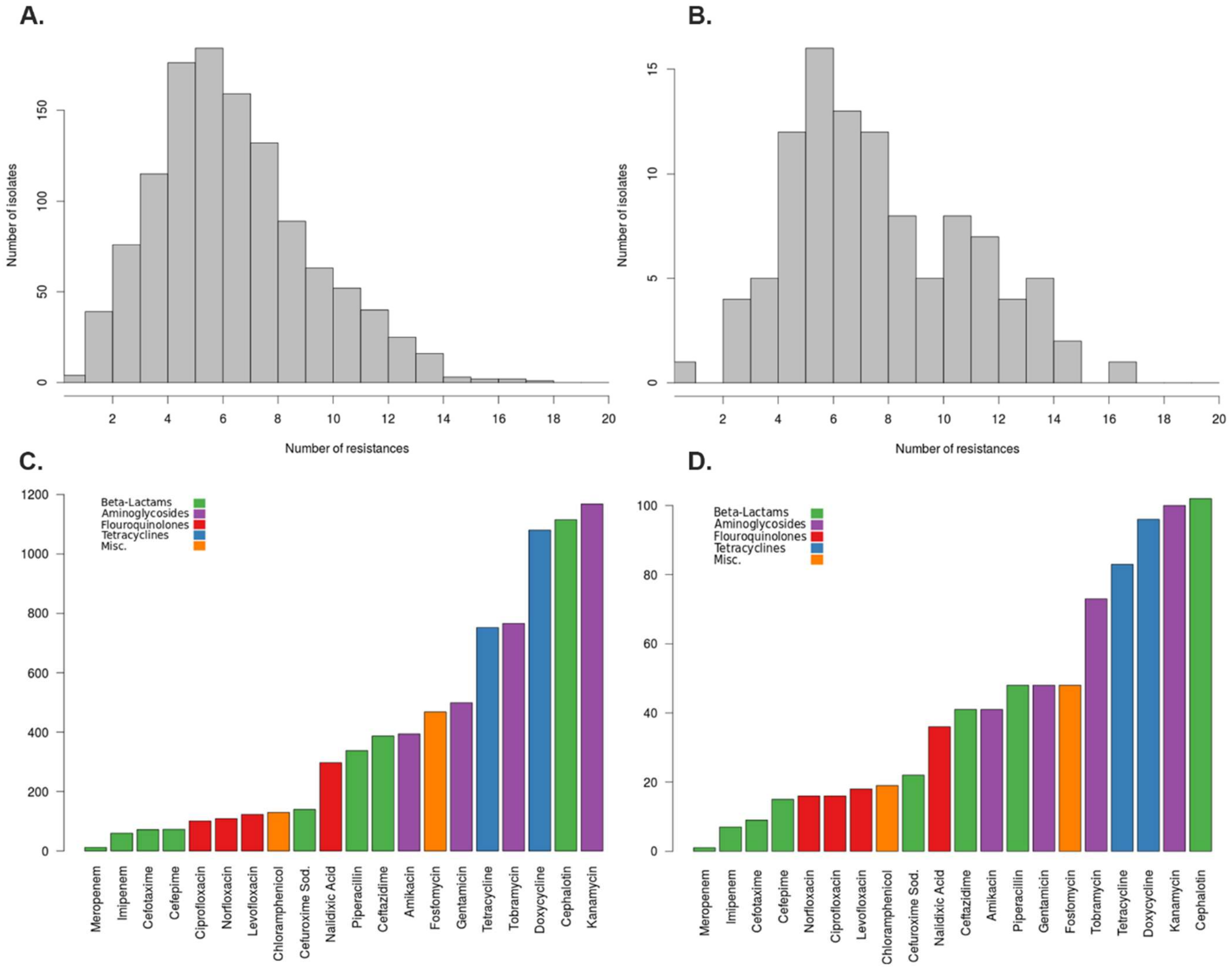

Figure 1: A and B. Number of resistances vs. number of isolates for all 1178 isolates (A) and the subset selected for sequencing (B). Both distributions are roughly equal but B has slightly more resistances. Overall, there are isolates resistant to 18 of twenty antibiotics. C and D. Antibiotics vs. number of resistant isolates for all 1178 isolates (C) and the subset selected for sequencing (D). Order among antibiotics is roughly equal between A and B. Beta-Lactams and Fluoroquinolones have fewest resistances.

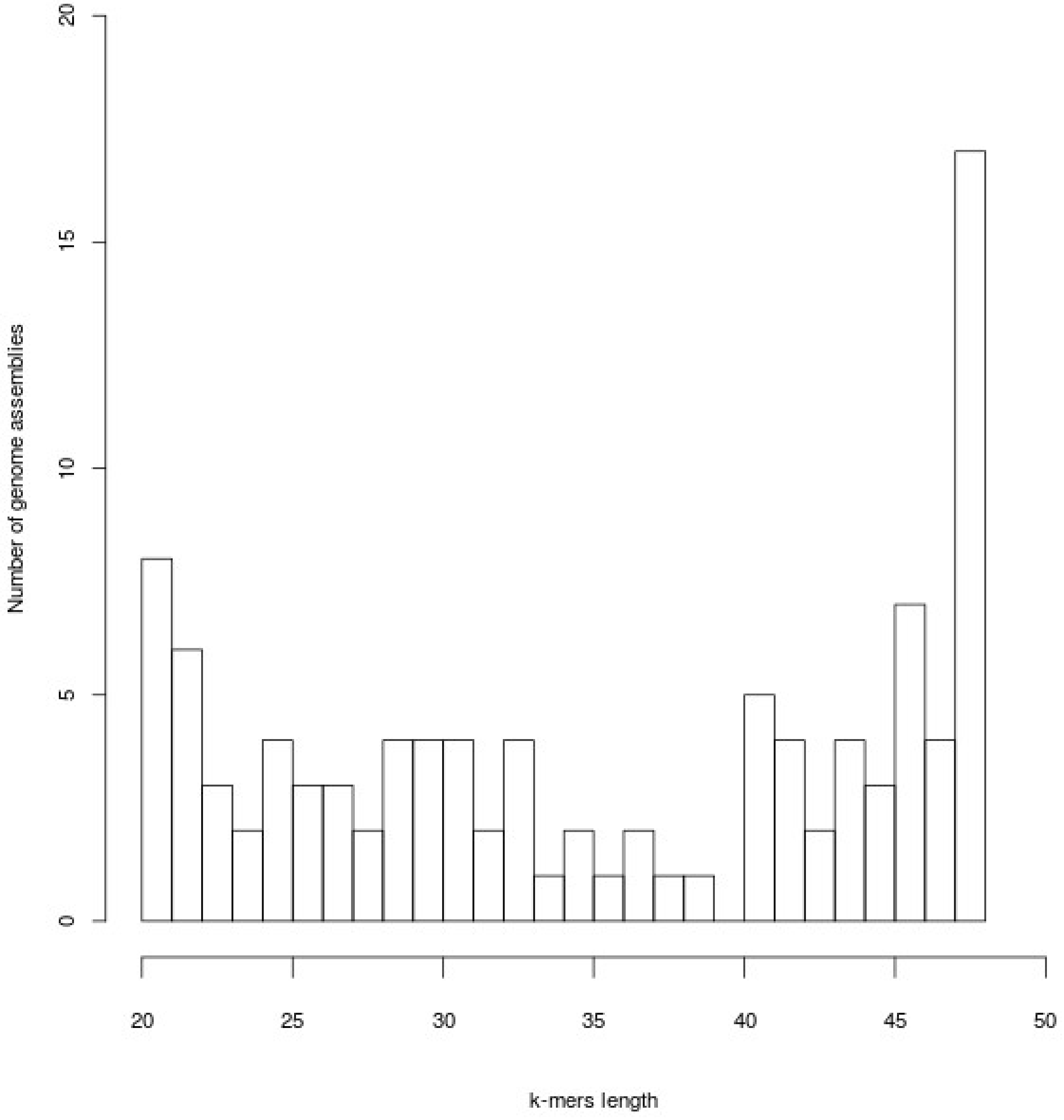

Figure 2: 20 to 48-mers vs. Number of genome assemblies with maximal N50 values.

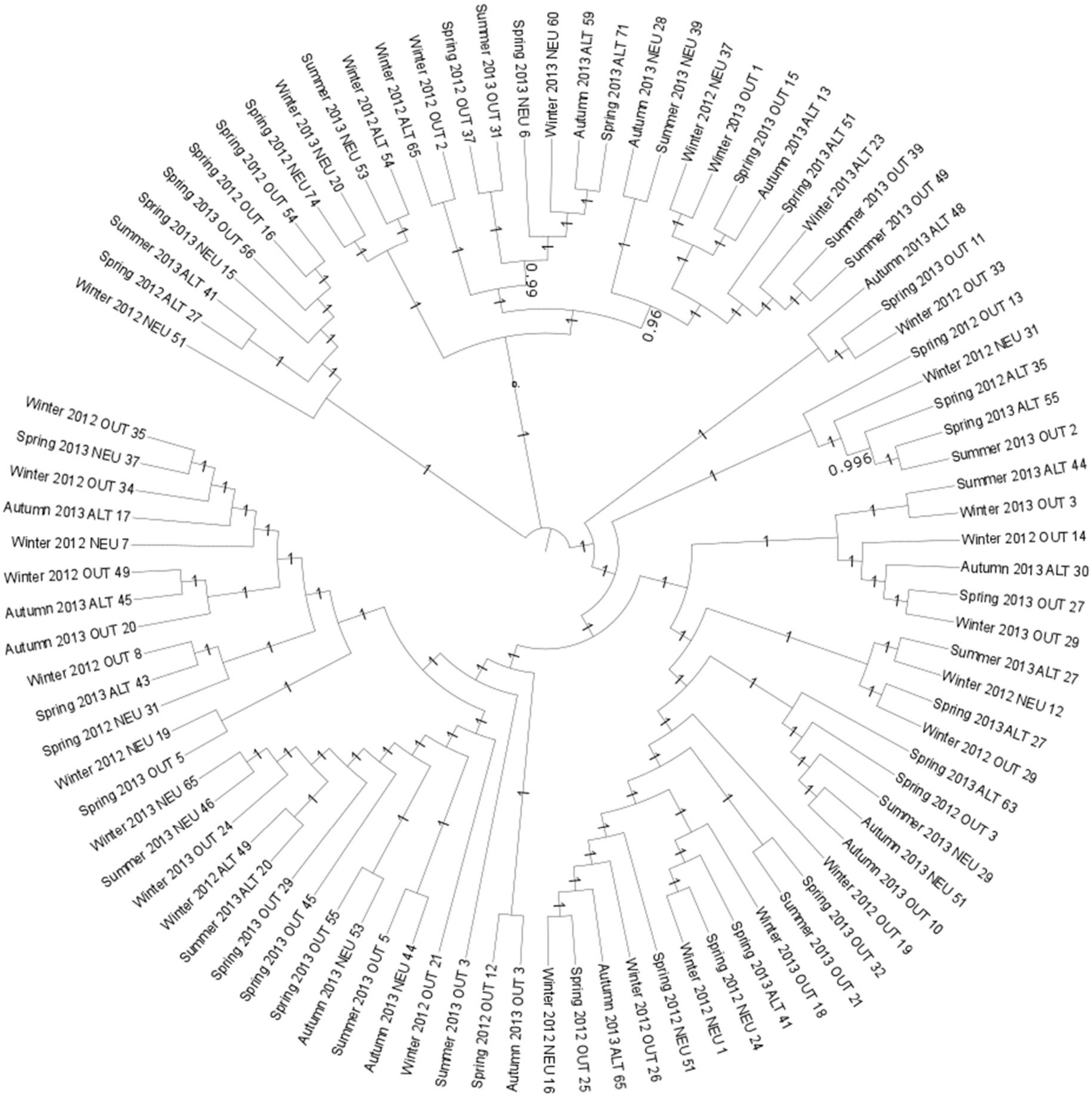

Figure 3: Phylogentic tree with bootstrap values.

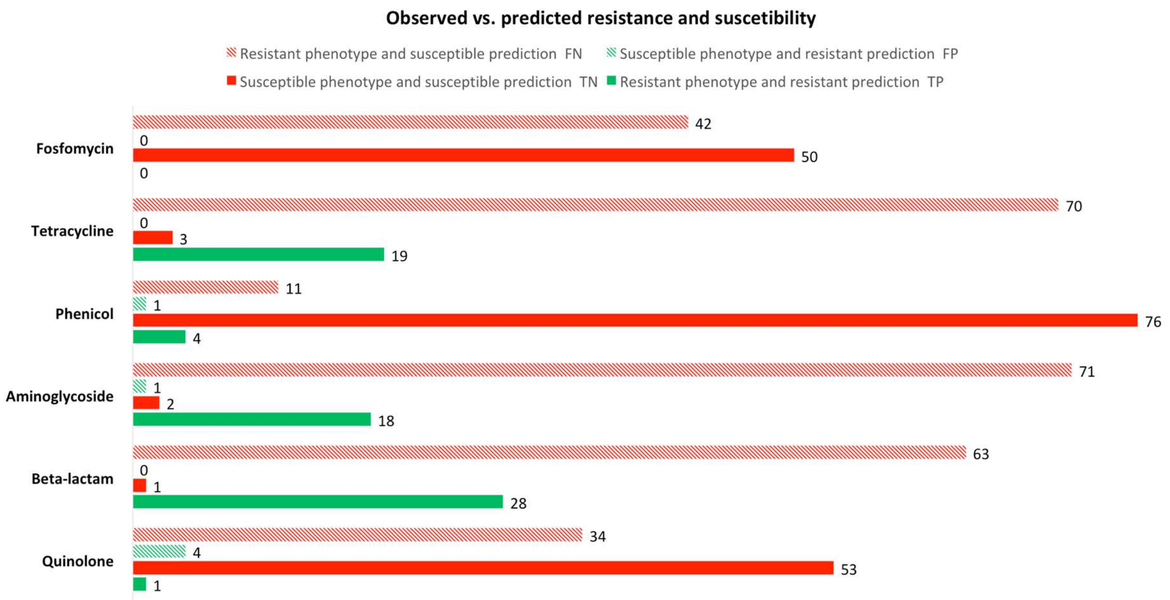

“Figure 4. Observed vs. Predicted resistance/susceptibility. Bold bars are correct predictions (True negatives TN and True positives TP), shaded bars are false predictions (False positives FP and False negatives FN). Overall accuracy, is 46%.

ResFinder predicts resistance of isolates for the above classes of antibiotics. Since observed resistance was obtained for individual antibiotics, we considered an isolate resistant against a class of antibiotics if it was resistant against at least one antibiotic of that class. Otherwise the isolate was considered susceptible. Hence, if ResFinder predicts an isolate as resistant against (susceptible to) a class of antibiotics, then this was considered

- a true positive (true negative), if the isolate was observed to be resistant (susceptible) to at least one (all) antibiotics of that class and
- a false positive (false negative) if the isolate was observed to be susceptible (resistant) to all (at least one) antibiotics of that class.

Accuracy was computed as true positives and true negatives divided by total number of sequences. The analysis was carried with a sequence cut off of 98%. To ensure that the cut-off did not have a strong impact, the analysis was repeated with a sequence cut-off of 85% sequence identity. The results did not change. Isolates were submitted to ResFinder Oct 2015.”

Table **1**: 58 reference genomes available from NCBI for constructing the reference gene clusters

Name	Accession	Version	GI
Escherichia coli IAI1 chromosome, complete genome	NC_011741	NC_011741.1	218552585
Escherichia coli BL21(DE3) chromosome, complete genome	NC_012971	NC_012971.2	387825439
Escherichia coli 042, complete genome	NC_017626	NC_017626.1	387605479
Escherichia coli 536, complete genome	NC_008253	NC_008253.1	110640213
Escherichia coli 55989 chromosome, complete genome	NC_011748	NC_011748.1	218693476
Escherichia coli SMS-3-5 chromosome, complete genome	NC_010498	NC_010498.1	170679574
Escherichia coli ABU 83972 chromosome, complete genome	NC_017631	NC_017631.1	386637352
Escherichia coli B str. REL606 chromosome, complete genome	NC_012967	NC_012967.1	254160123
Escherichia coli APEC O1 chromosome, complete genome	NC_008563	NC_008563.1	117622295
Escherichia coli DH1, complete genome	NC_017638	NC_017638.1	387619774
Escherichia coli APEC O78, complete genome	NC_020163	NC_020163.1	443615330
Escherichia coli ATCC 8739 chromosome, complete genome	NC_010468	NC_010468.1	170018061
Escherichia coli CFT073 chromosome, complete genome	NC_004431	NC_004431.1	26245917
Escherichia coli IAI39 chromosome, complete genome	NC_011750	NC_011750.1	218698419
Escherichia coli BL21(DE3), complete genome	NC_012892	NC_012892.2	387823261
Escherichia coli BW2952 chromosome, complete genome	NC_012759	NC_012759.1	238899406
Escherichia coli DH1 chromosome, complete genome	NC_017625	NC_017625.1	386593590
Escherichia coli IHE3034 chromosome, complete genome	NC_017628	NC_017628.1	386597751
Escherichia coli HS, complete genome	NC_009800	NC_009800.1	157159467
Escherichia coli ETEC H10407, complete genome	NC_017633	NC_017633.1	387610477
Escherichia coli ED1a chromosome, complete genome	NC_011745	NC_011745.1	218687878
Escherichia coli O103:H2 str. 12009, complete genome	NC_013353	NC_013353.1	260842239
Escherichia coli str. K-12 substr. DH10B chromosome, complete genome	NC_010473	NC_010473.1	170079663
Escherichia coli KO11FL chromosome, complete genome	NC_017660	NC_017660.1	386698504
Escherichia coli str. K-12 substr. MDS42 DNA, complete genome	NC_020518	NC_020518.1	471332236
Escherichia coli JJ1886, complete genome	NC_022648	NC_022648.1	556550243
Escherichia coli str. K-12 substr. MG1655, complete genome	NC_000913	NC_000913.3	556503834
Escherichia coli KO11FL chromosome, complete genome	NC_016902	NC_016902.1	378710836
Escherichia coli O111:H- str. 11128, complete genome	NC_013364	NC_013364.1	260866153
Escherichia coli str. K-12 substr. W3110, complete genome	NC_007779	NC_007779.1	388476123
Escherichia coli LF82, complete genome	NC_011993	NC_011993.1	222154829
Escherichia coli LY180, complete genome	NC_022364	NC_022364.1	544388862
Escherichia coli O157:H7 str. EDL933 chromosome, complete genome	NC_002655	NC_002655.2	16445223
Escherichia coli O127:H6 str. E2348/69 chromosome, complete genome	NC_011601	NC_011601.1	215485161
Escherichia coli O104:H4 str. 2009EL-2071 chromosome, complete genome	NC_018661	NC_018661.1	407466711
Escherichia coli NA114 chromosome, complete genome	NC_017644	NC_017644.1	386617516
Escherichia coli S88 chromosome, complete genome	NC_011742	NC_011742.1	218556939
Escherichia coli O104:H4 str. 2011C-3493 chromosome, complete genome	NC_018658	NC_018658.1	407479587
Escherichia coli O104:H4 str. 2009EL-2050 chromosome, complete genome	NC_018650	NC_018650.1	410480139
Escherichia coli O157:H7 str. TW14359 chromosome, complete genome	NC_013008	NC_013008.1	254791136
Escherichia coli O83:H1 str. NRG 857C chromosome, complete genome	NC_017634	NC_017634.1	387615344
Escherichia coli PMV-1 main chromosome, complete genome	NC_022370	NC_022370.1	544574430
Escherichia coli O55:H7 str. CB9615 chromosome, complete genome	NC_013941	NC_013941.1	291280824
Escherichia coli UMNK88 chromosome, complete genome	NC_017641	NC_017641.1	386612163
Escherichia coli O26:H11 str. 11368 chromosome, complete genome	NC_013361	NC_013361.1	260853213
Escherichia coli UM146 chromosome, complete genome	NC_017632	NC_017632.1	386602643
Escherichia coli O7:K1 str. CE10 chromosome, complete genome	NC_017646	NC_017646.1	386622414
Escherichia coli SE15, complete genome	NC_013654	NC_013654.1	387828053
Escherichia coli P12b chromosome, complete genome	NC_017663	NC_017663.1	386703215
Escherichia coli SE11 chromosome, complete genome	NC_011415	NC_011415.1	209917191
Escherichia coli Xuzhou21 chromosome, complete genome	NC_017906	NC_017906.1	387880559
Escherichia coli UTI89 chromosome, complete genome	NC_007946	NC_007946.1	91209055
Escherichia coli UMN026 chromosome, complete genome	NC_011751	NC_011751.1	218703261
Escherichia coli str. 'clone D i2' chromosome, complete genome	NC_017651	NC_017651.1	386627502
Escherichia coli 'BL21-Gold(DE3)pLysS AG' chromosome, complete genome	NC_012947	NC_012947.1	253771435
Escherichia coli W chromosome, complete genome	NC_017664	NC_017664.1	386707734
Escherichia coli W chromosome, complete genome	NC_017635	NC_017635.1	386607309
Escherichia coli str. 'clone D i14' chromosome, complete genome	NC_017652	NC_017652.1	386632422
